# A retrospective study to identify risk factors for somnolence and dizziness in patients treated with pregabalin

**DOI:** 10.1186/s40780-015-0022-7

**Published:** 2015-07-16

**Authors:** Hiroshi Kato, Masayuki Miyazaki, Mio Takeuchi, Hiroaki Tsukuura, Mihoko Sugishita, Yukihiro Noda, Kiyofumi Yamada

**Affiliations:** Department of Neuropsychopharmacology and Hospital Pharmacy, Nagoya University Graduate School of Medicine, 65 Tsurumai-cho, Showa-ku, Nagoya, Aichi 466-8560 Japan; Division of Clinical Sciences and Neuropsychopharmacology, Meijo University Faculty of Pharmacy, Nagoya, Japan; Department of Clinical Oncology and Chemotherapy, Nagoya University Hospital, Nagoya, Japan

**Keywords:** Pregabalin, Somnolence, Dizziness, Neuropathic pain, Cancer pain, Opioid, Risk factor

## Abstract

**Background:**

Pregabalin is a therapeutic drug for neuropathic pain that is associated with somnolence and dizziness. These adverse events are often experienced shortly after initiating pregabalin, and may lead to treatment discontinuation. The purpose of this study was to explore factors that influence the incidence of somnolence and dizziness induced by pregabalin, and to identify patients at higher risk of adverse events.

**Methods:**

A retrospective analysis was conducted of patient characteristics (age, gender, renal function, initial daily dose of pregabalin, co-administration of strong opioids and hypnotics) and the incidence of somnolence and dizziness during the first week of pregabalin treatment. An electronic chart was used to collect data from 204 inpatients prescribed pregabalin at Nagoya University Hospital from June 2011 to November 2012.

**Results:**

Among 36 patients who regularly received strong opioids, 18 (50.0 %) reported somnolence or dizziness during the first week of pregabalin treatment. The remaining 168 patients did not regularly receive strong opioids, and 25 (14.9 %) had an adverse event. In multivariate analysis, age (≧65 years, adjusted odds ratio: 2.507, 95 % CI: 1.164-5.397, *p* = 0.019) and regular co-administration of strong opioids (adjusted odds ratio: 5.507, 95 % CI: 2.460-12.328, *p* < 0.001) correlated with somnolence or dizziness.

**Conclusions:**

These data suggest that age (≧65 years) and co-administration of strong opioids are risk factors for somnolence or dizziness during pregabalin treatment for neuropathic pain. More careful dose titration is recommended for elderly patients and those receiving concomitant strong opioids.

## Background

The World Health Organization (WHO) analgesic ladder has been demonstrated to be effective for the treatment of cancer pain [1]. Patients with moderate-to-severe pain are candidates for starting strong opioid agents. However, because neuropathic cancer pain is resistant to opioids, certain adjuvant drugs are administered, including pregabalin, gabapentin, and duloxetine, and these have been shown to be effective [2–4].

Pregabalin is indicated for the management of neuropathic pain associated with diabetic peripheral neuropathy, postherpetic neuralgia, and spinal cord injury, and for the treatment of fibromyalgia. Pregabalin was first developed as an antiepileptic drug, and its efficacy for various types of neuropathic pain was demonstrated at a later time [5]. The efficacy of pregabalin for neuropathic pain has been attributed to modulation of glutamate release from hyperexcited neurons via binding to the alfa-2-delta (α2-δ) subunit of voltage-gated calcium channels, which modulates calcium influx to presynaptic terminals [6, 7]. Pharmacokinetic studies of pregabalin reveal that it is more than 90 % renally excreted as unchanged drug [8]. Accordingly, its clearance is reduced in patients with renal impairment [9].

The most common adverse events related to pregabalin treatment are somnolence and dizziness, which increase in a dose-dependent manner and represent the most frequent reasons for discontinuation of treatment [10–12]. Previous studies indicate that somnolence and dizziness associated with pregabalin therapy are transient, and most commonly reported in the first week of treatment, with a lower incidence thereafter [11–14]. Thus, careful dose titration to the highest tolerable dose is the most appropriate approach.

Neuropathic cancer pain is a frequent complication of cancer or its treatment, with approximately one-third of cancer pain cases involving a neuropathic component [15, 16]. Previous studies have demonstrated that adjuvant drugs, such as pregabalin, are efficacious for the treatment of neuropathic pain, although the majority of data have been obtained from non-cancer studies [17–20]. Recently, it has been reported that the neuropathic component of cancer pain responds more effectively to pregabalin than to strong opioids such as transdermal fentanyl [21]. Adjuvant drugs for the treatment of neuropathic cancer pain are included in recent clinical recommendations, usually as an add-on to opioids [22]. Therefore, cancer patients with pain who receive pregabalin treatment are often treated with opioids, which have inhibitory effects on the central nervous system. It is assumed that the combination of pregabalin with opioids may potentiate somnolence and dizziness.

The purpose of this study was to explore risk factors that influence the incidence of somnolence and dizziness induced by pregabalin, and to identify patients at higher risk of adverse events who require more careful administration of pregabalin.

## Methods

### Study procedures

This retrospective study was approved by the ethical committee of Nagoya University Hospital. Inpatients who were prescribed pregabalin (Lyrica® capsule) at Nagoya University Hospital from June 2011 to November 2012 were included. Exclusion criteria included patients who had initiated pregabalin treatment as an outpatient or in other hospitals, and presented somnolence, dizziness, or consciousness disorder caused by medication or electrolyte imbalance such as hypercalcemia at the time starting pregabalin treatment.

Patients’ baseline characteristics, including age, gender, renal function, neuropathic pain origin, initial dose of pregabalin (mg per day), co-administration of strong opioids (morphine, oxycodone, and fentanyl, except when used solely for rescue), co-administration of hypnotics, and the incidence of somenolence and dizziness during the first week of pregabalin treatment were retrospectively examined using an electronic chart. Creatinine clearance (CLcr), as a measure of renal function, was evaluated using the Cockcroft-Gault equation as follows:

$$ \mathrm{CLcr}\ \left(\mathrm{mL}/ \min \right) = \left(140\ \hbox{-}\ \mathrm{age}\right) \times \mathrm{weight}\ \left(\mathrm{kg}\right)\ /\ 72 \times \mathrm{serum}\ \mathrm{creatinine}\ \left(\mathrm{mg}/\mathrm{dL}\right) \times 0.85\ \left(\mathrm{if}\ \mathrm{female}\right) $$

### Statistical analysis

To identify factors associated with the incidence of adverse events (somnolence or dizziness), univariate analysis was performed using age, gender, regular co-administration of strong opioids, renal function, initial dose of pregabalin, pregabalin dose group, and regular co-administration of hypnotics as independent variables. Factors with a p-value <0.05 in univariate analysis were evaluated as potential covariates in multivariate analysis using logistic regression modeling. All statistical analyses were performed using SPSS version 22 (SPSS Inc., USA). P-values <0.05 were considered to denote statistically significant differences.

## Results

### Patient characteristics

The study included 204 patients [male: 123 (60.3 %), female: 81 (39.7 %)] with a median age of 61 years (range: 18–92). Thirty-six patients (17.6 %) were regularly administered strong opioids (fentanyl: n = 17, oxycodone: n = 16, morphine: n = 3). The median dose of regular opioid (converted to oral morphine) was 60 mg/day (range: 15-300 mg/day). Initial dosages of pregabalin were 25 (n = 4), 50 (n = 3), 75 (n = 73), and 150 mg/day (n = 124) (Table 1).

**Table 1:** Patient characteristics (n = 204)

Age	
Median, year	61
Range, year	18-92
Gender	
Male, *n*(%)	123 (60.3)
Female , *n*(%)	81 (39.7)
Median CLcr^a^, mL/min	88.7
NP^b^ origin	
Cancer related NP, *n*(%)	55 (27.0)
Chemotherapy related NP, *n*(%)	24 (11.7)
Non-cancer related NP, *n*(%)	125 (61.3)
Regular strong opioid	
yes, *n*(%)	36 (17.6)
-fentanyl, *n*	17
-oxycodone, *n*	16
-morphine, *n*	3
Median dose^c^ (range), mg/day	60 (15–300)
no, *n*(%)	168 (82.4)
Initial dose of pregabalin	
150 mg/day, *n*(%)	124 (60.8)
75 mg/day, *n*(%)	73 (35.8)
50 mg/day, *n*(%)	3 (1.5)
25 mg/day, *n*(%)	4 (1.9)

^a^CLcr: creatinine clearance using the Cockcroft-Gault equation

^b^NP: neuropathic pain

^c^converted to oral morphine

### Relationship between renal function and adverse events

Initial doses of pregabalin according to renal function (CLcr) are depicted in Table 2. In patients with normal renal function (CLcr≧60 mL/min), a total daily dose of 150 mg pregabalin is recommended initially. In patients with impaired renal function, the total daily dose of pregabalin was reduced according to the degree of renal function (60 > CLcr≧30 mL/min: 75 mg/day, 30 > CLcr≧15 mL/min: 25–50 mg/day, 15 mL/min > CLcr: 25 mg/day).

**Table 2:** Distribution of initial daily doses of pregabalin according to renal function

Renal function	Initial dose of pregabalin (per day)			
	25 mg	50 mg	75 mg	150 mg
	(n = 4)	(n = 3)	(n = 73)	(n = 124)
CLcr^a^≧60	3	2	49	112
60 > CLcr≧30	0	1	20	11
30 > CLcr≧15	1	0	4	1
15 > CLcr	0	0	0	0

^a^CLcr: creatinine clearance (mL/min) using the Cockcroft-Gault equation

Based on the initial dosing recommendations for differing degrees of renal function in the prescribing information of Lyrica® capsules [10], patients were divided into three groups: "high dose group", "regular dose group", and "low dose group". The incidence of somnolence or dizziness in each group is presented in Table 3. Seven (43.8 %) of 16 patients in the high dose group, 28 (21.1 %) of 133 patients in the regular dose group, and 8 (14.5 %) of 55 patients in low dose group reported somnolence or dizziness.

**Table 3:** Adverse events according to pregabalin dose group

Pregabalin dose group	Adverse event^a^, n		Incidence of adverse event, %
	(+)	(−)	
High	7	9	43.8
Regular	28	105	21.1
Low	8	47	14.5

^a^Adverse event: somnolence or dizziness

### Univariate analysis

Table 4 displays the relationship between each factor (age, gender, regular co-administration of strong opioids, renal function (CLcr), initial dose of pregabalin, pregabalin dose group, and regular co-administration of hypnotics) and incidence of adverse events (somnolence or dizziness) determined by univariate analysis. Among patients who regularly used strong opioids with pregabalin, 18 (50.0 %) of 36 exhibited somnolence or dizziness during the first week of pregabalin therapy. In contrast, adverse events were reported by only 25 (14.9 %) of the 168 patients who did not regularly receive strong opioids. Age (≧65 years), regular co-administration of strong opioids, renal function (CLcr < 60 mL/min), and high pregabalin doses were related to adverse events. Among these factors, regular co-administration of strong opioids had the highest odds ratio (odds ratio: 5.720, 95 % CI: 2.624-12.470, *p* < 0.001).

**Table 4:** Univariate analysis

Factor		Adverse event^a^		Odds ratio	95 % CI	*P*-value
		(+)	(−)			
Age	≧65	26	55	2.948	1.474-5.892	0.002
	<65	17	106			
Gender	male	31	92	1.937	0.928-4.044	0.078
	female	12	69			
Regular strong opioid	yes	18	18	5.720	2.624-12.470	<0.001
	no	25	143			
CLcr	<60 mL/min	13	25	2.357	1.083-5.133	0.031
	≧60 mL/min	30	136			
Initial dose of pregabalin	150 mg/day	30	94	1.645	0.799-3.387	0.177
	<150 mg/day	13	67			
Pregabalin dose group	high	7	9	3.284	1.146-9.407	0.027
	regular/low	36	152			
Hypnotics	yes	8	20	1.611	0.655-3.962	0.299
	no	35	141			

^a^Adverse event: somnolence or dizziness

### Multivariate analysis

The results of multivariate analysis are presented in Table 5. Independent variables included: age, regular co-administration of strong opioids, renal function (CLcr), and pregabalin dose group. The dependent variable was incidence of adverse events (somnolence or dizziness). Age (≧65 years, adjusted odds ratio: 2.507, 95 % CI: 1.164-5.397, *p* = 0.019) and regular co-administration of strong opioids (adjusted odds ratio: 5.507, 95 % CI: 2.460-12.328, *p* < 0.001) were significantly associated with the incidence of somnolence or dizziness.

**Table 5:** Multivariate analysis

Factor		Adjusted odds ratio	95 % CI	*P*-value
Age	≧65	2.507	1.164-5.397	0.019
Regular strong opioid	yes	5.507	2.460-12.328	<0.001
CLcr	<60 mL/min	1.138	0.380-3.409	0.817
Pregabalin dose group	high	2.172	0.518-9.099	0.289

## Discussion

Data from this study suggest that age and co-administration of regular strong opioids are risk factors for somnolence or dizziness in patients treated with pregabalin. Elderly patients often present with impairments in drug metabolism and excretion. Pregabalin undergoes renal excretion as an unchanged drug without hepatic metabolism [8, 23]. However, in the present multivariate analysis, renal impairment was not associated with the occurrence of somnolence and dizziness. In an analysis of patient-level data from 31 clinical trials of pregabalin, the risk of somnolence and dizziness was higher in elderly patients compared with that of other adverse events (for example, weight gain and dry mouth) [11]. This suggests that elderly patients on pregabalin therapy may have a higher risk of central nervous system adverse events such as somnolence and dizziness, but these events may not be due to renal impairment.

It has been reported that the AUC (area under the blood concentration curve) of gabapentin, a similar drug to pregabalin, is elevated when gabapentin is co-administrated with morphine [24]. This is explained by the increased absorption of gabapentin because of morphine-induced gastrointestinal hypomotility. The pharmacokinetic properties of pregabalin are different from those of gabapentin. Pregabalin has greater than 90 % bioavailability, regardless of dosage, while the bioavailability of gabapentin decreases as the dose increases. Therefore, the extent of gastrointestinal absorption of pregabalin, which has high bioavailability, may not be altered by concomitant opioid administration. Pharmacodynamic, but not pharmacokinetic interaction could be suggested in co-administration of pregabalin and opioid. Pregabalin is structurally related to gamma-aminobutyric acid (GABA), but it does not bind to GABA_A_, GABA_B_, or benzodiazepine receptors [6]. Gabapentin maintains and potentiates GABAergic systems, and it suppresses neuronal activity by increasing GABA levels in the brain [25, 26]. Thus, it is possible that the modulating effects of pregabalin on GABAergic systems influence the sedative effects of opioids in an additive manner, exacerbating somnolence and dizziness. However, co-administration of hypnotics was not associated with the incidence of somnolence and dizziness significantly in our study.

In a previous report, the impact of disease conditions on the incidence of pregabalin-associated adverse events was analyzed [11]. The incidence of somnolence and dizziness in cancer patients with neuropathic pain who received pregabalin treatment was not different from that observed for other diseases such as diabetic peripheral neuropathy and postherpetic neuralgia. Although no clear relationship was observed between cancer and the incidence of somnolence and dizziness in that report, administration of pregabalin to patients on regular strong opioids increased the incidence of these adverse events according to our data.

Previously, it has been suggested that the incidence of somnolence and dizziness in patients treated with pregabalin increases in a dose-dependent manner [11, 12]. However, in the present study, the initial dose of pregabalin did not have a significant impact on adverse event rates. Differences between studies include longer observation periods of several weeks, and more variable maintenance doses of pregabalin (maximum: 600 mg/day). Thus, it is likely that the initial dose of pregabalin has minimal impact on the incidence of somnolence and dizziness.

Furthermore, high pregabalin doses were not associated with a higher incidence of somnolence or dizziness, using the initial dose recommendations based on renal function. Although the initial dose of pregabalin was adjusted according to renal function, some patients received high or low doses of pregabalin. Under such conditions, dose of pregabalin did not have a significant impact on adverse events. This aspect may need to be studied for a longer observation period in a larger sample size using high doses. In the present study, the observation period was restricted to one week and the sample size was small (high dose group; n = 16).

The present study has limitations, including the retrospective, single-center design. Further prospective studies with a larger sample size are needed. Also, effect of hypnotics prescribed as needed is not taken into account in this study because of the limited data.

## Conclusions

In conclusion, these findings suggest that age (≧65 years) and co-administration of strong opioids are risk factors for somnolence or dizziness in patients on pregabalin treatment. Thus, more careful dose titration of pregabalin for neuropathic pain therapy is recommended in elderly patients, and those who receive strong opioids concomitantly.

## References

[1] World Health Organization (1986). Cancer Pain Relief.

[2] Vondracek P, Oslejskova H, Kepak T, Mazanek P, Sterba J, Rysava M, Gal P (2009). Efficacy of pregabalin in neuropathic pain in paediatric oncological patients. Eur J Paediatr Neurol..

[3] Mishra S, Bhatnagar S, Goyal GN, Rana SP, Upadhya SP (2012). A comparative efficacy of amitriptyline, gabapentin, and pregabalin in neuropathic cancer pain: a prospective randomized double-blind placebo-controlled study. Am J Hosp Palliat Care..

[4] Matsuoka H, Makimura C, Koyama A, Otsuka M, Okamoto W, Fujisaka Y, Kaneda H, Tsurutani J, Nakagawa K (2012). Pilot study of duloxetine for cancer patients with neuropathic pain non-responsive to pregabalin. Anticancer Res..

[5] Bennett MI, Laird B, van Litsenburg C, Nimour M (2013). Pregabalin for the management of neuropathic pain in adults with cancer: a systematic review of the literature. Pain Med..

[6] Taylor CP, Angelotti T, Fauman E (2007). Pharmacology and mechanism of action of pregabalin: the calcium channel alpha2-delta (alpha2-delta) subunit as a target for antiepileptic drug discovery. Epilepsy Res..

[7] Dooley DJ, Taylor CP, Donevan S, Feltner D (2007). Ca2+ channel alpha2delta ligands: novel modulators of neurotransmission. Trends Pharmacol Sci..

[8] Corrigan BW, Pool WF, Posvar EL, Strand JC, Alvey CW, Radulovic LL, Bockbrader HN (2001). Metabolic disposition of pregabalin in healthy volunteers [abstract PI-68]. Clin Pharmacol Ther.

[9] Randinitis EJ, Posvar EL, Alvey CW, Sedman AJ, Cook JA, Bockbrader HN (2003). Pharmacokinetics of pregabalin in subjects with various degrees of renal function. J Clin Pharmacol..

[10] Pfizer Inc. Lyrica US Prescribing Information. http://labeling.pfizer.com/ShowLabeling.aspx?id=561 (2013). Accessed 28 March 2015.

[11] Freynhagen R, Serpell M, Emir B, Whalen E, Parsons B, Clair A, Latymer M (2015). A Comprehensive Drug Safety Evaluation of Pregabalin in Peripheral Neuropathic Pain. Pain Pract..

[12] Gil-Nagel A, Zaccara G, Baldinetti F, Leon T (2009). Add-on treatment with pregabalin for partial seizures with or without generalisation: pooled data analysis of four randomised placebo-controlled trials. Seizure..

[13] Toelle TR, Varvara R, Nimour M, Emir B, Brasser M (2012). Pregabalin in Neuropathic Pain Related to DPN, Cancer and Back Pain: Analysis of a 6-Week Observational Study. Open Pain J..

[14] Saif MW, Syrigos K, Kaley K, Isufi I (2010). Role of pregabalin in treatment of oxaliplatin-induced sensory neuropathy. Anticancer Res..

[15] de Paredes ML G, del Moral González F, Martínez del Prado P, Martí Ciriquián JL, Enrech Francés S, Cobo Dols M (2011). First evidence of oncologic neuropathic pain prevalence after screening 8615 cancer patients. Results of the On study. Ann Oncol.

[16] Irving GA (2005). Contemporary assessment and management of neuropathic pain. Neurology.

[17] Rosenstock J, Tuchman M, LaMoreaux L, Sharma U (2004). Pregabalin for the treatment of painful diabetic peripheral neuropathy: a double-blind, placebo-controlled trial. Pain..

[18] Dworkin RH, Corbin AE, Young JP, Sharma U, LaMoreaux L, Bockbrader H, Garofalo EA, Poole RM (2003). Pregabalin for the treatment of postherpetic neuralgia: a randomized, placebo-controlled trial. Neurology..

[19] Siddall PJ, Cousins MJ, Otte A, Griesing T, Chambers R, Murphy TK (2006). Pregabalin in central neuropathic pain associated with spinal cord injury: a placebo-controlled trial. Neurology..

[20] Arnold LM, Russell IJ, Diri EW, Duan WR, Young JP, Sharma U, Martin SA, Barrett JA, Haig G (2008). A 14-week, randomized, double-blinded, placebo-controlled monotherapy trial of pregabalin in patients with fibromyalgia. J Pain..

[21] Raptis E, Vadalouca A, Stavropoulou E, Argyra E, Melemeni A, Siafaka I (2014). Pregabalin vs. opioids for the treatment of neuropathic cancer pain: a prospective, head-to-head, randomized, open-label study. Pain Pract.

[22] Ripamonti CI, Santini D, Maranzano E, Berti M, Roila F, Group EGW (2012). Management of cancer pain: ESMO Clinical Practice Guidelines. Ann Oncol.

[23] Brodie MJ (2004). Pregabalin as adjunctive therapy for partial seizures. Epilepsia.

[24] Eckhardt K, Ammon S, Hofmann U, Riebe A, Gugeler N, Mikus G (2000). Gabapentin enhances the analgesic effect of morphine in healthy volunteers. Anesth Analg..

[25] Petroff OA, Hyder F, Rothman DL, Mattson RH (2000). Effects of gabapentin on brain GABA, homocarnosine, and pyrrolidinone in epilepsy patients. Epilepsia..

[26] Cai K, Nanga RP, Lamprou L, Schinstine C, Elliott M, Hariharan H, Reddy R, Epperson CN (2012). The impact of gabapentin administration on brain GABA and glutamate concentrations: a 7T ^1^H-MRS study. Neuropsychopharmacology..

