# Erratum to: A retrospective study to identify risk factors for somnolence and dizziness in patients treated with pregabalin

**DOI:** 10.1186/s40780-016-0039-6

**Published:** 2016-02-17

**Authors:** Hiroshi Kato, Masayuki Miyazaki, Mio Takeuchi, Hiroaki Tsukuura, Mihoko Sugishita, Yukihiro Noda, Kiyofumi Yamada

**Affiliations:** Department of Neuropsychopharmacology and Hospital Pharmacy, Nagoya University Graduate School of Medicine, 65 Tsurumai-cho, Showa-ku, Nagoya, Aichi 466-8560 Japan; Division of Clinical Sciences and Neuropsychopharmacology, Meijo University Faculty of Pharmacy, Nagoya, Japan; Department of Clinical Oncology and Chemotherapy, Nagoya University Hospital, Nagoya, Japan

## Erratum

Unfortunately, after publication of the original version of this article [1], it was noticed that some author corrections were not included in the final article.

- Within the “Methods” section, the Cockcroft-Gault equation should read as follows:
  - $$ \mathrm{CLcr}\ \left(\mathrm{mL}/ \min \right) = \left(\mathbf{140} - \mathrm{age}\right) \times \mathrm{weight}\ \left(\mathrm{kg}\right)\ /\ \left\{\mathbf{72} \times \mathrm{serum}\ \mathrm{creatinine}\ \left(\mathrm{mg}/\mathrm{dL}\right)\right\} \times \mathbf{0.85}\ \left(\mathrm{if}\ \mathrm{female}\right) $$
- In addition Table 1 contained some formatting errors. The correct version of Table 1 is included below.

**Table 1:** Patient characteristics (n = 204)

Age	
Median, year	61
Range, year	18-92
Gender	
Male, *n*(%)	123 (60.3)
Female , *n*(%)	81 (39.7)
Median CLcr^a^, mL/min	88.7
NP^b^ origin	
Cancer related NP, *n*(%)	55 (27.0)
Chemotherapy related NP, *n*(%)	24 (11.7)
Non-cancer related NP, *n*(%)	125 (61.3)
Regular strong opioid	
yes, *n*(%)	36 (17.6)
-fentanyl, *n*	17
-oxycodone, *n*	16
-morphine, *n*	3
Median dose^c^ (range), mg/day	60 (15–300)
no, *n*(%)	168 (82.4)
Initial dose of pregabalin	
150 mg/day, *n*(%)	124 (60.8)
75 mg/day, *n*(%)	73 (35.8)
50 mg/day, *n*(%)	3 (1.5)
25 mg/day, *n*(%)	4 (1.9)

^a^CLcr: creatinine clearance using the Cockcroft-Gault equation

^b^NP: neuropathic pain

^c^converted to oral morphine
